# Supplementary figures and images for: A previously overlooked, highly diverse early Pleistocene elasmobranch assemblage from southern Taiwan
Source: PeerJ. 2022 Oct 20;10:e14190. doi: 10.7717/peerj.14190 (PMC9588305; doi:10.7717/peerj.14190)

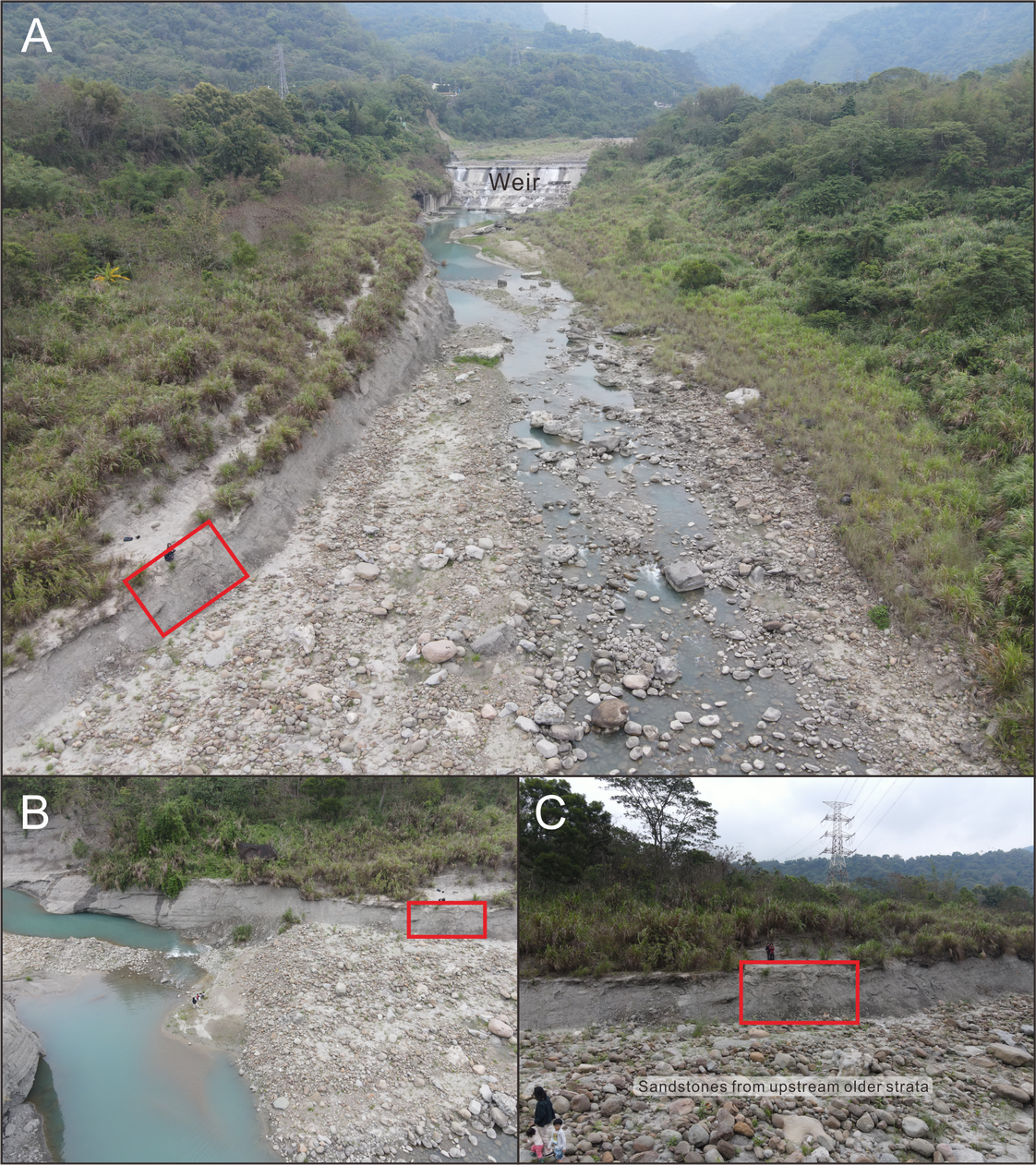

Supplement: Figure S1 — A, general view; B, C, closer views of the sampling site. Note the sandstones in the river bed transported from the upstream older strata. [file peerj-10-14190-s001.png]
